# Symmetric Level Index Arithmetic in Simulation and Modeling

**DOI:** 10.6028/jres.097.020

**Published:** 1992

**Authors:** Daniel W. Lozier, Peter R. Turner

**Affiliations:** National Institute of Standards and Technology, Gaithersburg, MD 20899; U.S. Naval Academy, Annapolis, MD 21402

**Keywords:** computer graphics, generalized logarithms and exponentials, least-squares data-fitting, overflow, underflow, and scaling, parallel computing, symmetric level-index arithmetic

## Abstract

This paper begins with a general introduction to the symmetric level-index, SLI, system of number representation and arithmetic. This system provides a robust framework in which experimental computation can be performed without the risk of failure due to overflow/underflow or to poor scaling of the original problem. There follows a brief summary of some existing computational experience with this system to illustrate its strengths in numerical, graphical and parallel computational settings. An example of the use of SLI arithmetic to overcome graphics failure in the modeling of a turbulent combustion problem is presented. The main thrust of this paper is to introduce the idea of SLI-linear least squares data fitting. The use of generalized logarithm and exponential functions is seen to offer significant improvement over the more conventional linear regression tools for fitting data from a compound exponential decay such as the decay of radioactive materials.

## 1. Introduction

In the field of scientific computation generally and especially in experimental computing which is often at the heart of simulation and modeling problems, the availability of a robust system of arithmetic offers many advantages. Many such computational problems are prone to failure due to overflow or underflow or to a lack of advance knowledge of a suitable scaling for the problem. The use of a computer arithmetic system which is free of these drawbacks would clearly alleviate any such difficulties.

One such arithmetic is the symmetric level index, SLI, system. (See [3] for an introductory summary.) This system was developed from the original level-index system of Clenshaw and Olver [1] and has been studied in a number of subsequent papers. Working to any finite precision, it is closed under the four basic arithmetic operations (apart from division by zero, of course) and is therefore free of underflow and overflow. The arithmetic system allows very large or very small numbers which may not be representable in a conventional floatingpoint system to be used during interim computation while still returning meaningful results.

Section 2 of the paper describes, briefly, the SLI representation and its basic algorithms and properties. The natural error measure for SLI arithmetic, generalized precision, is also introduced. This measure will be used in the SLI-linear least squares data fitting experiments described in Sec. 4.

In Sec. 3, a brief summary of some of the existing computational evidence supporting the use of SLI arithmetic is presented. This concentrates first on two examples (the robust computation of binomial probabilities and the solution of polynomial equations by the root-squaring technique) which demonstrate the ability of the system to recover valuable information from interim results which would exceed the range of any floating-point system. The other principal example reviewed here is taken from the modeling of turbulent combustion. In this case, it is seen that commercial contour plotting packages used with the floating-point system produce seriously misleading (but initially plausible) results while the true situation is revealed by the use of SLI arithmetic. In this case there is no straightforward rescaling of the original problem which would allow successful floatingpoint computation.

In Sec. 4, we concentrate on a topic which is commonly used in simulation and statistical analysis–least squares data-fitting. Our interest is in curve-fitting, *not* the related problem of parameter estimation. The particular problem discussed is a compound exponential decay such as might be encountered in modeling the decay of radioactive materials. Compound decays with widely varying half-lives are not well approximated by the commonly used log-linear least squares method. The use of the SLI representation function–a generalized logarithm–permits much better agreement with the data while still using very low degree approximating functions. The benefits derived from using SLI arithmetic–or, in this case, just the SLI representation–are made plain by a sequence of graphical examples.

## 2. The SLI Arithmetic System

The level-index number system for computer arithmetic was first suggested in Clenshaw and Olver [1], [2]. The scheme was extended to the symmetric level index, SLI, representation in [4] and has been studied in several further papers in the last few years. Much of the earlier work is summarized in the introductory survey [3]. The primary virtue of SLI arithmetic is its freedom from overflow and underflow and the consequent ease of algorithm development available to the scientific software designer. This is not the only arithmetic system that has been proposed with this aim: for example, the work of Matsui and Iri [14], Hamada [7], [8], and Yokoo [27] suggested and studied modified floating-point systems which share some properties of level-index; Smith, Olver, and Lozier [21] studied an extended range arithmetic.

Possible hardware implementations of SLI arithmetic were discussed in [18], [23], and [26] while a software implementation incorporating some extended arithmetic was described in [25]. The error analysis of SLI arithmetic is discussed in [2] and [4] and is extended in [11], [16], and [17]. Applications and software engineering aspects of the level-index system have been discussed in [5], [10], [12], and [24].

The SLI representation of a real number *X* is given by

$$X=s_{x}\phi {\left(x\right)}^{r_{x}}$$

where the two signs *s_x_* and *r_x_* are ± 1 and the *generalized exponential* function is defined for *x* ⩾ 0 by

$$\phi \left(x\right)=\{\begin{array}{cc} x & 0⩽x\;<1,\\ \exp\;\left(\phi \left(x-1\right)\right) & x>1. \end{array}$$

It follows that for *X* > 1,

X=exp(exp(…(expf)…))

where the exponentiation is performed *l*=[*x*] times and *x=l+f.* The integer part *l* of *x* is called the *level* and the fractional part *f* is called the *index.*

The freedom of this system from over- and underflow results from the fact that, working to a precision of no more than 5 million binary places in the index, the system is closed under the four basic arithmetic operations with only three bits allotted to the level. This is discussed briefly in [1], [4] and considered in some detail in [11].

The basic SLI arithmetic operation is that of finding the SLI representation $s_{z}\phi {\left(z\right)}^{r_{z}}$ of *Z* = *X* ± *Y* where *X, Y* are also given by their SLI representations. Without loss of generality, we may assume that *X* ⩾ *Y* > 0 so that *s_z_* = + l. The computation entails the calculation of the members of three short sequences which vary according to the particular circumstances. In every case, the sequence defined by

$$a_{j}=\frac{1}{\phi \left(x-j\right)}\;\left(j=l-1,\;l-2,\ldots ,0\right)$$

where *l* = [*x*], is computed using the recurrence relation

$$a_{j-1}=\exp\left(-1/a_{j}\right);\;a_{l-1}=e^{-f}.$$

Depending on the values of *r_x_, r_y_*, and *r_z_*, the other sequences that may be required are given, for appropriate starting values, by

$$\begin{array}{l} b_{j}=\frac{\phi \left(y-j\right)}{\phi \left(x-j\right)},\;\beta_{j}=\frac{\phi \left(x-j\right)}{\phi \left(y-j\right)},\;\alpha_{j}=\frac{1}{\phi \left(y-j\right)}\\ c_{j}=\frac{\phi \left(z-j\right)}{\phi \left(x-j\right)},\;h_{j}=\phi \left(z-j\right). \end{array}$$

Specifically, the sequence (*b_j_*) is used when *r_y_* =* +* 1. The terms can be computed using

$$b_{j-1}=\exp\;\left(\frac{b_{j}-1}{a_{j}}\right)$$

with the initial value given by

$$b_{m-1}=a_{m-1}\;e^{g}=\{\begin{array}{ll} \exp\;(g-1/a_{m}) & \left(m<l\right)\\ \exp\;(g-f) & \left(m=l\right) \end{array}$$

where *m* =[*y*] is the level of y. Since, in this case, *y⩽x*, it follows that 0⩽*b_j_* <1.

The sequence (*α_j_*) is used when *r_x_* = +1, *r_y_* =* −*1 and is computed like (*α_j_*). It is similarly bounded: 0*⩽α_j_*, *α_j_<l.*

For the case where *r_x_* =*r_y_* =* −*1, the requirement *X*⩾*Y* implies *x⩽y*. The sequence (*α_j_*) is computed as before along with the sequence (*β_j_*). This latter is computed using the recurrence relation

$$\beta_{j-1}=\exp\left(\frac{\beta_{j}-1}{a_{j}\beta_{j}}\right)$$

for *j* <*l* with the initial value

$$\beta_{l-1}=\{\begin{array}{ll} \exp\;(f-1/\alpha_{l}) & \left(l<m\right)\\ \exp\;(f-g) & \left(l-m\right) \end{array}.$$

The results of these calculations can be combined to yield, for the first two cases

$$c_{0}=1\pm b_{0}\;\mathrm{or}\;c_{0}=1\pm a_{0}\alpha_{0}$$

while for the “small” case, we compute

$$c_{0}^{\prime }=\frac{1}{c_{0}}=1\pm \beta_{0}$$

from which the required terms of the *c*-sequence may be computed by the recurrence

$$c_{j}=1+a_{j}\;\ln\;c_{j-1}.$$

The number of such terms is bounded by *l* – 1 after which some terms of the sequence *h_j_* may be needed. This just involves forming repeated natural logarithms of *c_l_*_–1_. Detailed derivations of the various sequences can be found in [2] and [4].

This algorithm is implemented in the software implementation of SLI arithmetic described in [25]. This implementation also takes advantage of the relative ease of performing extended arithmetic operations such as summation or forming scalar products in SLI arithmetic, the details of which are discussed more fully in [25], [26]. The major advantage of these extended operations is likely to be in parallel computing environments where most of the likely speed loss suffered by SLI arithmetic will be recouped.

The results of [26] indicate that a parallel SLI processor could yield a reasonable speed-up over serial floating-point computation for extended operations of even moderate length. A fairer “parallel-parallel” comparison suggests a likely slowdown of arithmetic by a factor of around 2 for extended operations–which probably represents a loss of some 10 to 20% in run-time. However, even that small price to pay for a robust arithmetic is likely to be recouped for many parallel operations.

In [12], it is seen that several basic operations such as forming vector norms are difficult to program both efficiently and robustly for floating-point machines but become straightforward tasks in the SLI environment. It is the simplicity of programming, and code which is free of special scaling or exception-handling cases, which are likely to tip the balance in favor of SLI arithmetic once suitable hardware implementations exist.

The appropriate error measure for computation in the level index system is no longer relative error (which corresponds approximately to absolute precision in the mantissa of floating-point numbers) but *generalized precision* which corresponds to absolute precision in the index. This error measure is introduced in [1]. Generalized precision has some significant advantages compared to relative error, not least of which is that it is a metric so that the symmetry of *x* approximating $\bar{x}$, and $\bar{x}$ approximating *x*, is a natural aspect of the error analysis. Detailed error analyses of numerical processes will inevitably be different in this system than for any of the floating-point systems but significant progress has already been made in this respect. (See, for example, [1], [17], [26].)

Again considerable benefits can be achieved for extended calculations. Olver [17] has demonstrated that, at relatively low cost, it is possible to perform a concurrent error analysis. Such analysis is particularly well-suited to a parallel environment since it would be performed by simultaneous duplication of the operations for slightly adjusted data. The adjustments are similar to the use of directed rounding in interval arithmetic and have a similar effect in yielding guaranteed error bounds for the results obtained.

A first-order error analysis for extended sums and products [26] leads to conclusions that are broadly similar to those for the floating-point system. However, for the SLI system, we find that the generalized precision of the final result is bounded by *N*/2 times the generalized precision for individual operations.

## 3. Computational Evidence

We give three examples of computations which are highly susceptible to underflow and/or overflow, and we present computational evidence that SLI arithmetic produces valid results. The first example, the binomial probability distribution, was treated in [22] to introduce and evaluate techniques for dealing with underflow and overflow in floating-point, FLP, computation. The second example, the classical Graeffe algorithm for determining the zeros of a polynomial, is almost never used in FLP computation because the root-squaring process almost always introduces very large and/or very small numbers as intermediate results. The final example is drawn from a computer graphics display of an analytical solution to a model problem in turbulent combustion theory.

### 3.1 Binomial Probability Distribution (BPD)

In addition to [22] this example was treated in [14] and [3]. The BPD and its importance in statistics is discussed, for example, in [15], pp. 54–58. Let *p* be the probability of a favored outcome from an individual chance event. Then *q* = 1–*p* is the probability that the favored outcome will not occur. Now, the probability of the favored outcome occurring exactly *k* times in *n* events is

$$f_{k}=({\;}_{k}^{n})p^{k}q^{n-k}.$$

This distribution attains its maximum when *k* equals its *modal value*

$$k_{c}=\left[\left(n+1\right)p\right]$$

and *f_k_* decreases steadily as *k* moves away from *k_c_.*

The values of *f_k_* are exceedingly small when *n is* of moderate size and *k* is not close to *k_c_* For example, [22] considers *n* =2000 and *p* =0.1. Since the underflow threshold^1^ in IEEE standard 32-bit FLP arithmetic is 1.18×10^−38^ for normalized numbers, *f_k_* underflows when 0⩽*k*⩽51 and 393⩽*k*⩽2000. Replacement of these underflows by zero (or de normalized numbers) may be acceptable for many purposes, such as for the computation of the cumulative probability

$$I\left(n,m,p\right)=\sum_{k=0}^{m}f_{k}\;\left(0\;⩽\;m\;⩽\;n\right).$$

In reality, however, *f_k_* and *I* are always strictly positive, and it can be disconcerting when a computer program prints zero.

A more serious problem, perhaps, is that an FLP algorithm must guard carefully against intermediate underflow and overflow. Overflow is possible in forming the binomial coefficient

$$\left(\begin{array}{c} n\\ k \end{array}\right)=\frac{n\left(n-1\right)\ldots \left(n-k+1\right)}{k!}$$

and underflow is possible in forming powers of *p* and *q.* Indeed, with *n* =2000, *p* =0.1 and *k =k_c_* = 200,

$$f_{200}=0.02972\;287717$$

but

$$\left(\begin{array}{c} n\\ k \end{array}\right)=10^{280.84},\;p^{200}=10^{-200},\;q^{1800}=10^{-82.36}$$

Since 0 <*f_k_* < 1, the algorithm cannot be allowed to fail because of overflow. Zero is an acceptable result only if *f_k_* is below the underflow threshold.

Two algorithms are considered in [22]. Algorithm I forms *f_k_* by (i) multiplying in all factors of the numerator of the binomial coefficient, (ii) dividing out all factors of the denominator, (iii) multiplying in all factors of *p^k^*, and (iv) multiplying in all factors of *q^n–k^.* As we have seen, this algorithm is sure to fail in many cases of interest. It is rendered usable in [22] by introducing a counter *l* and two large positive constants *c*_1_ and *c*_2_ such that *c*_1_*c*_2_ is slightly less than the overflow threshold and 1/*c*_1_*c*_2_ is slightly more than the underflow threshold. Let us refer to this modification as Algorithm IA. Before each arithmetic operation, Algorithm IA tests the current result. If it does not lie between 1/*c*_1_ and *c*_1_, it is scaled into this interval by multiplying or dividing by *c*_1_ and incrementing or decrementing *l* accordingly. At the end, $c_{1}^{I}f_{k}$ is represented as a normalized FLP number with *l* ⩾0. If *l* = 0, the algorithm is complete. If *l* = 1 and *c*_1_*f_k_*<l/*c*_2_, the algorithm divides once by *c*_1_ and returns *f_k_*. In all other cases the algorithm returns zero.

Algorithm II forms *f_k_* from the same operations as Algorithm I but in different order. Operations from part (i) increase the current result, whereas those from the other three parts decrease it. Algorithm II uses only a single large constant *c*_1_. It starts with increasing operations. When the current result would exceed *c*_1_, Algorithm II switches to decreasing operations until the current result would fall below 1/c_1_, at which point it switches back to increasing operations. The algorithm ends when either *f_k_* is completely formed or the current result falls below 1/*c*_1_ and no increasing operations remain, in which case *f_k_* is returned as zero.

These algorithms may be compared as follows. Algorithm I produces the shortest program with the simplest logic. It requires no tests or system-dependent constants. It is feasible for SLI but not for FLP arithmetic. Algorithm IA produces a program more than twice as long because, at every arithmetic operation, it requires a test and contingent coding to handle underflow or overflow. The number of contingent operations depends on *p, n, k, c*_1_ and *c*_2_. The latter two constants depend on the underflow and overflow thresholds but there is no natural mathematical definition of this dependence. A counter is required also. The algorithm is designed for FLP and is not appropriate for SLI arithmetic. Algorithm II produces a program that is slightly more complex and slightly shorter than Algorithm IA. It requires a test but no contingent coding at every arithmetic operation, only one constant, and no counter. It is feasible for both FLP and SLI arithmetic.

Table 1 summarizes these observations. The table also gives some data on the relative error in the computed value of *f*_200_, with *p* =0.1 and *n* =2000, in 32-bit SLI and FLP arithmetic. Measured relative errors were obtained by comparison against 64-bit FLP calculations. The measured error is similar for Algorithm II in both arithmetics, with the smaller error occurring in SLI. For Algorithm I, the FLP error is ∞ because of overflow failure. The SLI error is larger than for Algorithm II because the simpler algorithm generates larger intermediate values (up to 10^655.7^ for Algorithm I, 10^363^ for Algorithm II). Algorithm IA is not appropriate for SLI and was used only as a specific remedy for FLP arithmetic. It performed 31 contingent operations in computing *f*_200_ and produced an error slightly larger than the FLP error of Algorithm II.

### 3.2 The Graeffe Root-Squaring Process

At the end of the preceding subsection, it was suggested that one algorithm for the BPD leads to larger relative errors than another because the first algorithm generates larger intermediate values. Although this is true for the BPD, it is not a reliable guide for all algorithms. Indeed, it is easily proved that the relative error in a function *y =f*(*x*) caused by a relative error its argument is approximated to first order by

$$\delta y≃A_{f}\left(x\right)\delta x$$

where

$$A_{f}\left(x\right)=\frac{xf^{\prime }\left(x\right)}{f\left(x\right)}.$$

This function is sometimes called the *relative-error amplification factor* for *f*. However, it either amplifies or deamplifies the relative error according to whether |*A_f_*(*x*)| > 1 or |*A_f_*(*x*)| < 1. If |(*A_f_*(*x*)|⪡1, the deamplification effect is very strong. An example is *y =x^a^* for 0*<a* <1. Here the deamplification factor is *A_f_*(*x*)≡*a*.

The Graeffe root-squaring process is a very old numerical method for solving algebraic equations; in [9] it is traced back to 1762. For more modern accounts, see [6], [9], and [20], pp. 1174–8. Let

$$p(x)=a_{n}x^{n}+a_{n-1}x^{n-1}+\ldots +a_{0},\;a_{n}\neq 0$$

be an arbitrary polynomial. If *γ* is a zero of *p*(*x*), the Graeffe process forms approximations to |*γ*|*^m^* where *m* can be arbitrarily large. More specifically, if a subset of zeros lies on or very near a circle in the complex plane, the method finds the *m*th power of the radius of the circle and an algebraic equation for the *m*th powers of the zeros in the subset. The size of *m* in any particular application depends on how well separated these circles are – the smaller the separation, the higher *m* must be. The idea of the method is to increase the separation of the circles by squaring their radii.

The Graeffe process involves the construction [20], p. 1174 of a finite number of the polynomials in the sequence

$$p^{\left(r\right)}\left(x\right)=a_{n}^{\left(r\right)}x^{n}+a_{n-1}^{\left(r\right)}x^{n-1}+\ldots +a_{0}^{\left(r\right)},\;a_{n}^{\left(r\right)}\neq 0$$

such that the zeros of *p*^(^*^r^*^)^(*x*) are the zeros of *p*(*x*) raised to the 2*^r^*th power. Here *p*^(0)^≡*p–*that is, $a_{j}^{\left(0\right)}=a_{j}$ for all *j* – and

$$a_{j}^{\left(r+1\right)}={\left(-1\right)}^{j}{\left[a_{j}^{\left(r\right)}\right]}^{2}+2\sum_{k=1}^{\min\left(j,n-j\right)}{\left(-1\right)}^{k}a_{j-k}^{\left(r\right)}a_{j+k}^{\left(r\right)}.$$

In particular,

$$a_{0}^{\left(r+1\right)}={\left[a_{0}^{\left(r\right)}\right]}^{2},\;a_{n}^{\left(r+1\right)}={\left(-1\right)}^{n}{\left[a_{n}^{\left(r\right)}\right]}^{2}.$$

As the iteration proceeds, some (or none or all) of the $a_{j}^{\left(r\right)}$ with 0*<j<n* begin to satisfy the approximation

$$a_{j}^{\left(r+1\right)}≃{\left(-1\right)}^{j}{\left[a_{j}^{\left(r\right)}\right]}^{2}.$$

Let us suppose this happens for some *r* and

$$0<j_{1}<j_{2}<\ldots <j_{s}<n$$

and let us define *j*_0_ = 0, *j_s+_*_1_=*n.* Then there are zeros of *p*(*x*) that lie on or very near a circle of radius

$$\left|\gamma_{i}\right|={\left|a_{ji}^{\left(r\right)}/a_{ji-1}^{\left(r\right)}\right|}^{2^{-r}}\left(i=1,2,\ldots s+1\right).$$

Because the deamplification factor 2^−^*^r^* is small, this determination of |*γ_i_*| should be very stable. This expectation is borne out by examples in [6], [9], and [20], all done by desk calculator or slide rule in the era before FLP computation became widespread. Indeed, on p. 187 of [6] it is stated that Graeffe’s method is “generally useful” and “well adapted to the computing machine or slide rule”; obviously the author did not appreciate the impact of underflow or overflow! In most of these examples numbers are generated that significantly exceed the IEEE overflow threshold, even in double precision.

When *s* = *n −*1, the preceding discussion suffices to summarize the use of the Graeffe process up to the determination of the phase angle; if the polynomial has all real coefficients, the phase angle is either 0 or *π* and the determination can be made by substitution of ± *γ_i_* into the original equation. In all other cases the method needs modification or extension. Some of these are indicated in [6], [9], and [20], including the important case of real polynomials with nonrepeated real and complex conjugate zeros.

The Graeffe process has fallen into disuse because it is not well suited to FLP computation. Certainly alternative methods are available to solve algebraic equations. One popular method is to use an algorithm from numerical linear algebra to find the eigenvalues of the so-called companion matrix of *p*(*x*). However, the Graeffe process could equally well be applied to the problem of finding matrix eigenvalues. This is a powerful motivation for considering SLI computer arithmetic.

Such a consideration was begun in [3] and [5], where the method is described and a preliminary error analysis is given. Several more substantial examples are computed in SLI arithmetic with very satisfactory results even when very large values of *m* were needed. The method has also received attention in [14].

### 3.3 Graphics for a Combustion Problem

A model problem in the theory of turbulent combustion, introduced in [13], involves two chemical species (fuel and oxidizer) which occupy two adjacent half-spaces separated conceptually by an impervious plane boundary. At time *t*=0, the boundary loses its imperviousness and a line vortex is imposed in the plane of the boundary, leaving the two species free to diffuse into each other, react, and produce a flame surface at the boundary, which is distorted by the vortex into a cylindrically symmetric spiral surface. With the axis of the vortex identified as the *z*-axis, cylindrical symmetry reduces the spatial variables to plane polar coordinates. The location and shape of the flame surface is of particular interest in applications. For example, reactant consumption and heat production are obtained by integrating the normal derivative of the solution along the flame surface.

This problem has undergone extensive analytical and computational development in recent years. For example, the development in [19] of a solution in terms of a similarity variable

$$\eta =\sqrt{\frac{r^{2}}{4vt}}$$

where *v* is the kinematic viscosity, opened up the possibility of a two-dimensional analysis.

Indeed, an explicit representation was obtained of a function *Z* (*η*, *θ*) as a Fourier series. The flame surface of the model problem corresponds to the level curves, or contours, of this function. Therefore, it can be computed (in principle, at least) by inverse interpolation from data on a grid. The data are the computed values of *Z* on the grid. Powerful graphics software is available to compute and display contours on a wide variety of graphics devices. Unfortunately, this software is not robust in the face of underflow, as we shall see in the case of the flame surface.

From a mathematical as well as a scientific standpoint, *Z*(*η*, *θ*) exhibits interesting behavior. As *η*→0 for fixed *θ*, it oscillates with unboundedly growing frequency while its magnitude tends to zero exponentially fast. Of particular interest is the shape of the contours *Z=±ϵ.* For a small *ϵ*>0, the two contours reflect each other in the origin. Their shape is a spiral which winds toward the origin up to a point that depends on *ϵ*, at which it abruptly reverses direction and winds away from the origin. In the limit as *ϵ*→0, the contours fuse into one that can be regarded as having two branches, one a spiral that winds right down to the origin while encircling it infinitely often and the other its reflection in the origin.

**Fig. 1:**
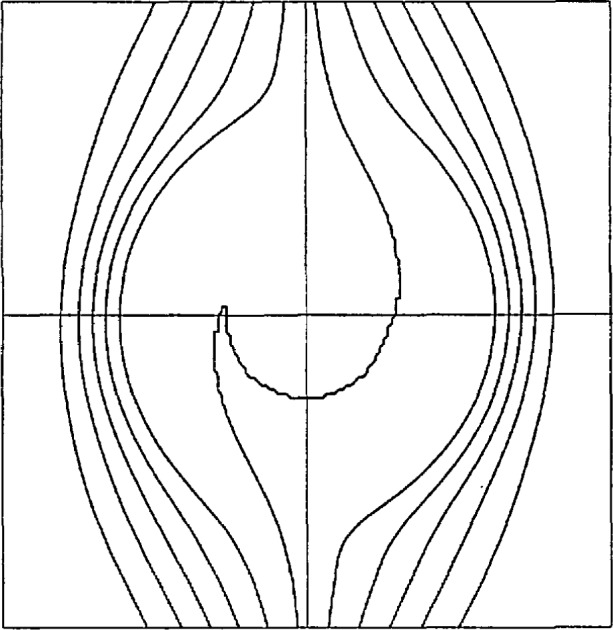
Contours of *Z* = −0.4(0.1)0.4 computed in IEEE single precision by the GCONTR graphics subroutine. Resolution = 151^2^.

This complicated behavior suggests that it would be a difficult challenge for graphics software to produce correct contours. Indeed, this is the case. IEEE single-precision arithmetic, with a word length of 32 bits, was used to execute a standard library subroutine for contour plotting. Fig. 1 shows the results for *ϵ* = 0. Clearly, the central contour does not exhibit the required symmetry or spiral behavior.

**Fig. 2:**
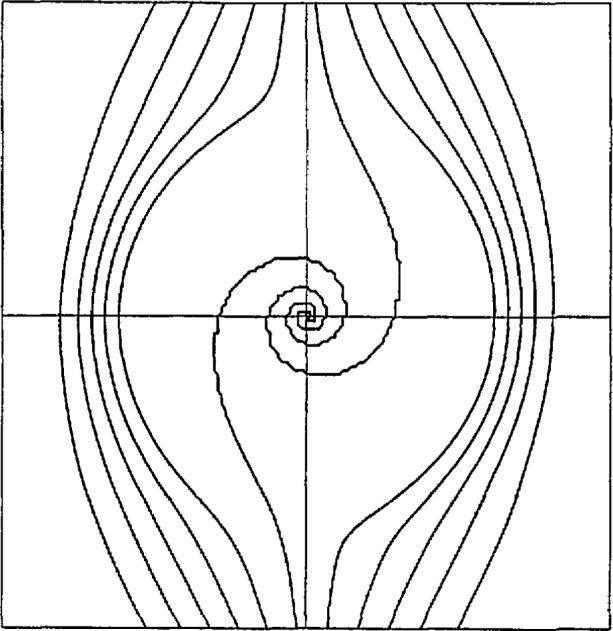
Contours of *Z* = −0.4(0.1)0.4 computed in 32-bit SLI by the GCONTR graphics subroutine. Resolution = 151^2^.

Figure 2 was produced in exactly the same way except SLI arithmetic was used. In particular, no algorithms were changed and no scaling was introduced. The mesh of 151^2^ points is identical. The smallest magnitude of data on the mesh, excluding the origin, was 10^−42800^, approximately. The contour shows the correct qualitative and quantitative behavior within the limits of the mesh resolution. With a refinement of the mesh, the infinite spiral would be resolved correspondingly closer to the origin; in fact, this was demonstrated on a mesh nine times finer, in which case the smallest magnitude was 10^−389985^, approximately.

**Fig. 3:**
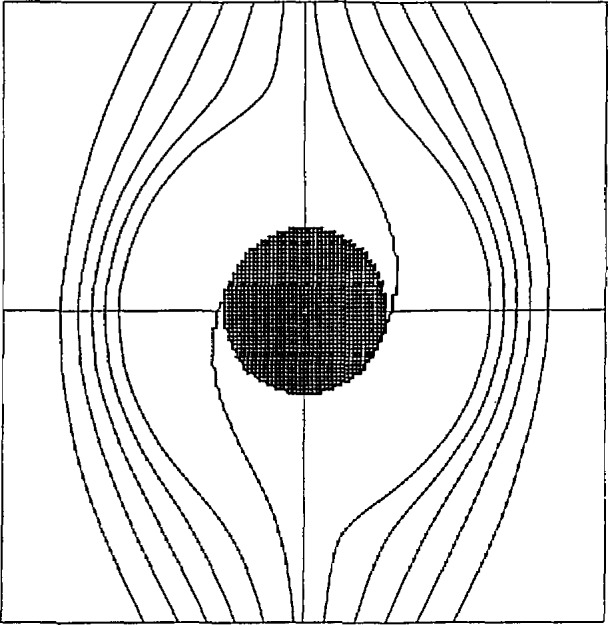
Contours of Z = −0.4(0.1)0.4 computed in IEEE single precision by the GCONTR graphics subroutine. Resolution = 151^2^. Black marks indicate points where *Z* underflowed.

Figure 3 is a repeat of Fig. 1 but with one modification: A black mark is plotted at every mesh point where the functional data was zero. The only mesh point where *Z* = 0 is the origin; all the other zeros are the result of underflow. This shows clearly that the sole cause of the graphics failure in FLP arithmetic is underflow.

From a scientist’s point of view, the meaning of these results is the following. Small values of *ϵ* correspond to cases of near stoichiometry among the concentrations of the combustion reactants. Perfect stoichiometry corresponds to complete combustion, in which all reactants are consumed fully. If a numerical experiment is conducted in which *ϵ* takes the sequence of values 10^−2^, 10^−4^, 0, say, the results are confusing. That is, the scientist sees correct behavior for the nonzero values but incorrect behavior for *ϵ* = 0. The transition to perfect stoichiometry cannot be made visible graphically with any currently available floating-point processor. This defect does not arise with SLI arithmetic.

We conclude this section by remarking that scaling does not provide an effective solution to the FLP graphics failure. The stoichiometric contour *Z* = 0 is obtained by inverse interpolation from nearby data on the grid. For grid points near the origin, as we have seen, this data is exceedingly small. However, for grid points near the boundary of the plotting region, the data is not small; certainly it is not below the underflow threshold. The data varies over some 40000 orders of magnitude. Since IEEE single precision varies over only 76 orders of magnitude, approximately 526 scalings would be required. Clearly, scaling attacks the failure only very weakly. Added to this weakness would be the difficulties presented by the contouring software itself. The scaling in this example needs to be done in thin annuli around the origin but the software works with rectangular regions. Therefore, a complicated mosaic of subregions would need to be combined to form the complete contour plot.

## 4. SLI-Linear Least Squares Data-Fitting

This section is concerned with a common technique in simulation – least squares fitting of data. The experiments reported provide a comparison between linear least squares, log-linear least squares and SLI-linear least squares as curve fitting techniques for data from compound exponential decay functions such as might arise in studying radioactive decay.

The principle of linear least squares polynomial fitting to data is to find that polynomial of fixed (maximum) degree, *N* say, for which the sum of squared errors

$$\sum_{i=0}^{n}{\left(y_{i}-p_{N}\left(x_{i}\right)\right)}^{2}$$

is minimized. Here, the data consists of the ordered pairs (*x_i_*,*y_i_*) and it is assumed that the number of data points *n +* 1 exceeds *N* (usually by a significant proportion).

The log-linear fit to this data uses the approximation exp(*q_N_* (*x*)) where *q_N_* is the least squares polynomial approximation to the data (*x_i_*,ln *y_i_*).

The SLI-linear fit uses *Φ*(*q_N_*(*x*)) where, this time, *q_N_* is the least squares polynomial approximation to the data (*x_i_*, *Ψ*(*y_i_*)) where the functions *Φ*, *Ψ* are the functions used in the SLI representation of positive real numbers. They are defined by:

$$\Psi \left(X\right)=\{\begin{array}{ll} \psi \left(X\right)-1 & X⩾1\\ 1-\psi \left(\frac{1}{X}\right) & X<1 \end{array}$$

and

$$\Phi \left(x\right)=\{\begin{array}{ll} \phi \left(1+x\right) & x⩾0\\ \phi {\left(1+\left|x\right|\right)}^{-1} & x<0 \end{array}$$

where *ϕ* is the generalized exponential defined in Sec. 2 and the generalized logarithm *ψ* is its inverse.

The first experiments all used the same test function to generate the data on [0, 5]:

$$f\left(x\right)=20e^{-5x}+5e^{-x}$$

which consists of two exponential decay components the first of which is initially much the greater but decays much faster. In all cases, the least squares approximations of the appropriate degree using polynomial, log-linear and SLI-linear fitting are plotted together with the original function for comparison.

**Fig. 4:**
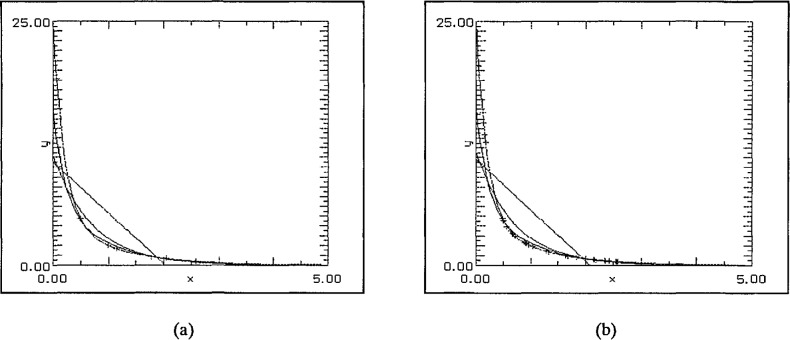
(a) 8 data points, and (b) 32 data points.

**Fig. 5:**
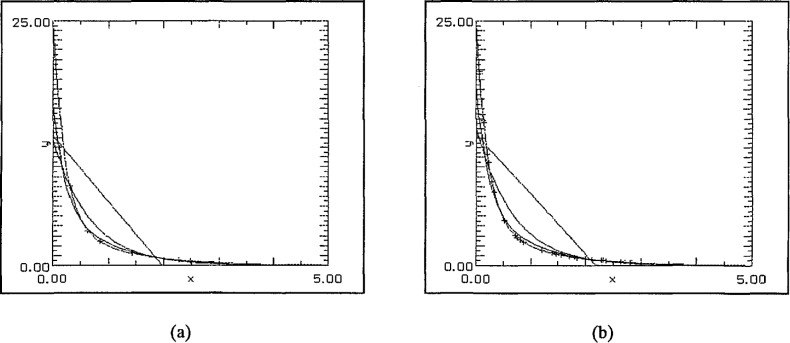
(a) 8 data points including 0, and (b) 32 data points including 0.

The first set of graphs, Fig. 4 uses (degree 1) linear least squares fits to randomly generated data sets in [0, 5] subject only to a bias towards zero in the selection. This was achieved by forcing approximately 50% of the data to lie in [0, 1], 25% in [1, 2], 12.5% in [2, 3] and so on. For the first pair of graphs the choice of data points was entirely random save for the distribution described above. The best fit was achieved with the SLI-linear approximation. In the second pair, Fig. 5, the further restriction that *x*_0_ = 0 was enforced. Again the SLI-linear approximation is the best in both cases and forcing 0 to be a data point has improved significantly the “goodness of fit” near the origin.

**Fig. 6:**
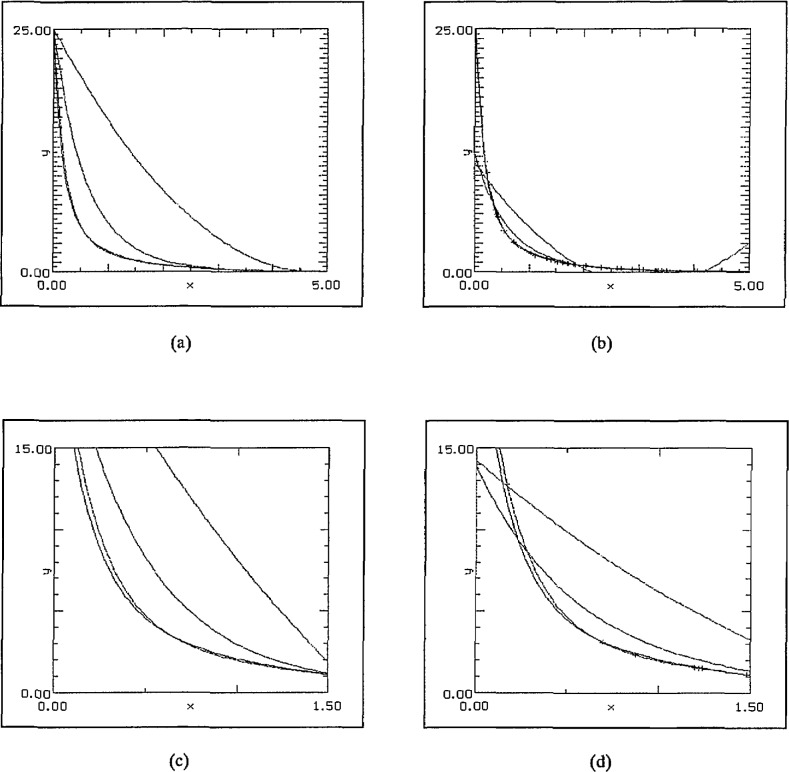
(a) 3 points, (b) 40 points, (c) 3 points, magnified view, and (d) 40 points, magnified view.

The next set of graphs, Fig. 6, shows the result of quadratic approximations using different numbers of randomly chosen data points with the constraint that two of them were tied to the end-points. In every instance two of the curves were almost indistinguishable: the test function and the SLI fit. The polynomial fit was uniformly the worst–as would be expected. Typically, the logarithmic fit either remains too high throughout the region or starts too low and then crosses to become too large. The second pair of graphs in Fig. 6 magnifies the region [0,1.5] × [0,15] for different random data sets.

**Fig. 7:**
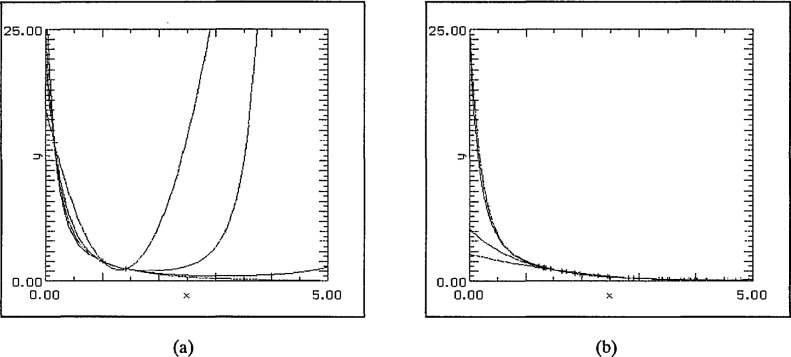
(a) 3 points, and (b) 35 points.

The effect of an entirely random data set is illustrated in Fig. 7. With even three data points the SLI-fit again produces tolerably good agreement with the original function–in sharp contrast to both the others having reached their respective minima to the left of *x* = 2. With a larger number of data points, the SLI approximation again reproduces the original function to high accuracy.

**Fig. 8:**
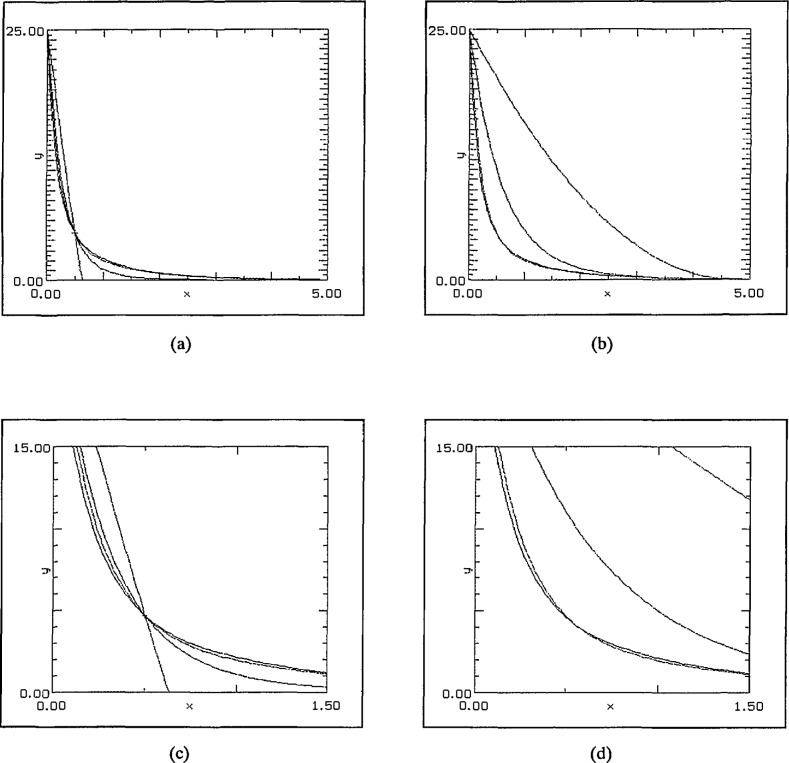
Three fixed data points: endpoints and stated point, (a) 0.5, (b) 4.5, (c) 0.5, magnified view, and (d) 4.5, magnified view.

The observation in the Figs. 6 and 7 that the SLI-quadratic fit using just three points (including the end-points) has produced such close agreement with the test function suggested further investigation to see whether this low degree approximation can be relied upon to produce high-accuracy approximations. This reliability persists for all choices of three data points {0, *i/*2, 5} for *i* = 1, 2, …, 9. The results for *i* = 1 and 9 are presented in Fig. 8. Again the second pair magnifies the region [0,1.5] × [0,15].

**Fig. 9:**
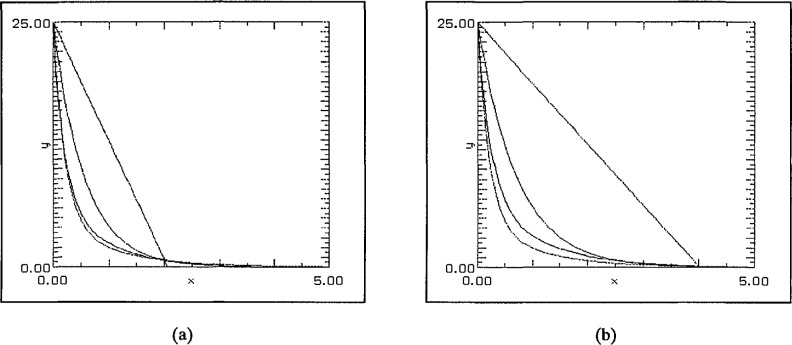
(a) Data points 0, 2, and (b) data points 0, 4.

Consideration of a table of differences suggests that interpolation at just two points may yield surprisingly good results. The evidence thus far is that use of the left-hand end-point is advantageous. This proved to be especially true in the linear interpolation case. Interpolation at 0 and *i* for *i* = 1,…, 5 continued the pattern of the SLI fit being visibly superior. The graphs for the cases *i* = 2 and 4 are reproduced in Fig. 9.

**Fig. 10:**
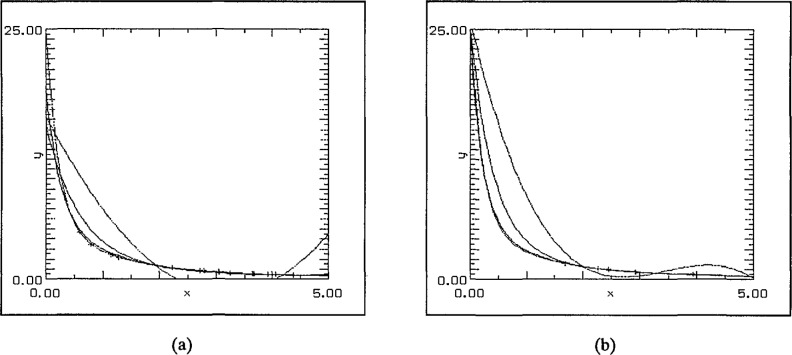
(a) 20 points, quadratic fit, and (b) 7 points, cubic approximation.

Experiments using 3 components including a term with much slower decay again had the consistent result that the SLI-approximations were superior. See Fig. 10. The data function used was:

$$f\left(x\right)=20e^{-5x}+5e^{-x}+{e^{-}}^{x/4}.$$

The first of the graphs in Fig. 10 shows the result of a quadratic approximation using 20 random data points including the end-points. The SLI approximation is almost exact except at the left-hand end where, like the others, it lies below the data curve. The second one demonstrates that increasing the degree of the approximation to cubic yields almost perfect agreement with a mere 7 data points. The polynomial fit has its local maximum around *x* = 4. The log-linear fit is also effective beyond about *x* = 2 by which stage the two faster decaying terms have become insignificant.

**Fig. 11:**
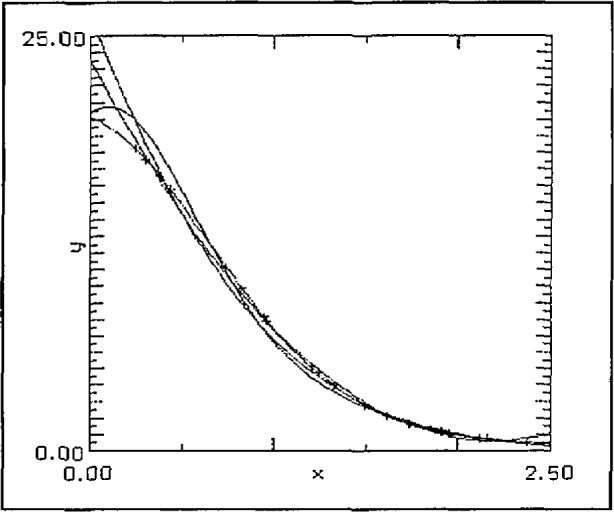
Faster than exponential decay, 20 random data points, quadratic approximations.

The final example was to approximate a “faster-than-exponential” decay given by

$$f\left(x\right)=25\exp\left(-x^{2}-1/2\right)+5\exp\left(-x\right)$$

over the interval [0, 2.5]. The graph is typical of the results which again indicate a superiority for the SLI-approximation. In this case quadratic approximations and 20 randomly placed data points were used. The SLI approximation is the only one with the right “shape” having an inflection point very close to that of the test function. The results are illustrated in Fig. 11.

## 5. Conclusions

The principal conclusions to be drawn from this paper are that SLI arithmetic offers a robust alternative to the floating-point system which enables many of the standard tasks of scientific computing to be performed in a simple yet reliable way. The computational evidence of Sec. 3 summarizes its power in three inherently different situations: computing binomial probabilities, solving polynomial equations and contour plotting for functions which have widely varying values and so are not amenable to scaling.

The final section discusses the use of SLI arithmetic in a curve-fitting situation and demonstrates that the SLI representation can be used to advantage in fitting data from a compound exponential decay. The experimental evidence indicates that a very good fit can be obtained with a low order SLI-linear least squares fit using only a small number of data points. This topic will be the subject of further experiment and analysis.

## Figures and Tables

**Table 1:** Attributes of three algorithms for the binomial probability distribution. *n* = number of events, *k* = number of favored outcomes, *c* = number of contingent operations to avoid underflow and overflow

	I	IA	II
Program length	Short	Long	Long
Program logic	Simple	Complex	Complex
No. constants	0	2	1
No. counters	0	1	0
No. operations	*n+*2*k*	*n+*2*k+c*	*n+*2*k*
No. tests	0	*n+*2*k*	*n+*2*k*
Measured rel err (SLI)	6.4×10^−4^	N.A.	−1.5×10^−5^
Measured rel err (FLP)	∞	−4.5×10^−5^	−4.3×10^−5^

## Footnotes

1 Here and elsewhere in this section, numbers written with a decimal point are correct to all places shown.
